# Age and sex related differences in shoulder abduction fatigue

**DOI:** 10.1186/s12891-018-2191-7

**Published:** 2018-08-07

**Authors:** John D. Collins, Leonard O’Sullivan

**Affiliations:** 1 School of Design, University of Limerick, Limerick, Ireland; 2 School of Design and Health Research Institute, University of Limerick, Limerick, Ireland

**Keywords:** Musculoskeletal disorders, Age, Sex, Trapezius, Shoulder, Fatigue

## Abstract

**Background:**

Injury prevalence data commonly indicate trends of higher rates of work-related musculoskeletal disorders in older workers over their younger counterparts, and for females more than males. The purpose of this study was to investigate age and sex-related differences in manifestations of shoulder muscle fatigue in a cohort of young and older working age males and females, in a single experiment design allowing for direct comparison of the fatigue effects between the target groups.

**Methods:**

We report upper trapezius muscle fibre Conduction Velocity (CV) as an indicative measure of muscle fatigability, and isometric endurance time, at three levels of shoulder abduction lifting force set relative to participants’ maximal strength.

**Results:**

Upper trapezius conduction velocity was significantly different between the young and old groups (*p* = 0.002) as well as between males and females (*p* = 0.016). Shoulder abduction endurance time was affected by age (*P* = 0.024) but not sex (*p* = 0.170).

**Conclusions:**

The study identified age-related improvement in muscle fatigue resistance and increased resistance for females over males, contrary to injury prevalence trends. The muscle fatigue effects are most likely explained by muscle fibre type composition. Experimental fatigue treatments of the upper trapezius were tested at exposures relative to the participants’ strength. Absolute strength is higher when young and is generally higher for males. The findings of this study point towards age and sex-related differences in strength rather than in muscle fatigue resistance as a primary cause for the differences in the injury trends.

## Background

Occupational injury and symptom prevalence data often indicate higher rates for older workers and for females [1, 2]. Collins and O’Sullivan [3] previously reported prevalence of neck/shoulder symptoms of Musculo-Skeletal Disorders (MSDs) for young and old age groups in a sedentary occupation, a trend also described by others [4]. In the study by Collins and O’Sullivan symptoms were highest for the oldest cohort and for females compared to their male counterparts. Silvia et al. [5] reported back pain across multiple industrial sectors and again reported higher prevalence for females. Laperriere et al. [6] detailed higher prevalence of self-reported work-related pain for females than males in food service work. Anton and Weeks [7] described higher rates of MSD symptoms for female grocery workers than their male counterparts. In addition, they reported higher rates for workers aged 35+ years compared to younger age groups. Regarding ageing and MSDs, Slovak et al. [8] analysed data from The Health and Occupational Reporting Network which indicated a fivefold increase in work-related musculoskeletal disorders from ages 15–24 to 45–64. This is of considerable concern in view of the ageing workforce [9]. Many occupational health and ergonomics studies of MSD prevalence focus on heavy manual work, yet MSDs are highly prevalent in low force sedentary work, specifically in relation to the shoulder, for example in computer-based work [2, 4, 10].

The authors propose that there are three primary explanations for age and sex-related differences in muscle-related occupational injuries. The first explanation is due to differences in exposures, where some occupational groups with higher risk of developing MSDs are dominated by one sex and/or age group. Controlling for exposure, a second potential explanation of variations are age/sex-related differences in muscle strength. A third possible explanation is due to differences in muscle fibre composition. The current study focuses on evaluating the latter explanation by measuring muscle fibre conduction velocity in a group of old and young participants.

Muscle activity, particularly where forceful exertions exist, has been implicated in many studies as a primary risk factor of muscle fatigue [11, 12]. However, muscle fatigue is not limited to high force contractions as low force static contractions can also cause muscle fatigue [13]. It is therefore important to acknowledge age and sex-based differences in fibre distribution as these characteristics may explain, at least in part, injury prevalence patterns detailed in previous research [3]. If age and sex-related differences in fibre types affect endurance and fatigue, it may be postulated that the magnitude of differences is important from an injury causation perspective.

There is good reason to consider muscle fibre-related differences as a mechanism of increased injury, especially for low force static contractions. The scientific literature on muscle fatigue resistance [14, 15] and muscle fibre type composition indicate age [16] and sex-based differences [17]. Furthermore, studies have investigated muscle fatigue and function specifically for low force muscle activity contractions, which is particularly important considering muscle fibre type differences [12, 13, 17]. According to the Hennmann Principle [18] and the Cinderella hypothesis [19], sustained low-level isometric contractions set up a stereotyped recruitment pattern of Motor Units (MUs) according to the size principle. Low threshold (type I) MUs are constantly active even in situations of continuous low muscle activity which could result in metabolically overloaded ‘Cinderella’ muscle fibres.

There are numerous studies of age and sex-related differences in muscle performance [14, 15]. However, we have been unable to find any studies specifically assessing age and sex-based differences in muscle fibre conduction velocity of the trapezius. The aim of this study was to investigate manifestations of shoulder muscle fatigue across age and sex. Conduction Velocity (CV) was used as an index of muscle fatigue in this experiment. It is possibly the most accurate physiological parameter of muscle fatigue as it is affected by fibre size and changes in pH, whereby CV values decrease during fatigue contractions and the slope of the CV values is indicative of the rate of fatigue in the muscle [20].

## Method

### Study design and statistical analysis

This was a laboratory based study of upper trapezius muscle fibre Conduction Velocity (CV) (as a measure of muscle fatigue and endurance) for three relative levels of shoulder abduction, tested with the shoulder abducted 90^0^. The two dependent variables were CV (normalised slope) and Endurance Time (ET). The independent variables were sex, age (young and old) and shoulder abduction exertion load (0, 10 and 20% Maximum Voluntary Contraction (MVC)). The exertion levels were based on previous studies of shoulder fatigue [21]. VO_2_ MAX was entered as a covariate to control for differences in aerobic fitness.

### Participants

There were 40 participants, 20 males and 20 females, with 10 each in the young and old sex-age groups (young participants mean age 26.0 years ±2.18 SD; older group mean age 59.6 years ±3.17 SD). A power analysis indicated this sample would yield an experimental power of > 0.8.

The experiment was approved by the University of Limerick Research Ethics Committee. Participants were recruited through advertisements on the University campus and through requests for volunteers through fellow researchers’ contacts. Participants gave their written informed consent prior to testing. No participant reported any known symptoms of locomotive or musculoskeletal disorders.

### Equipment

As muscle CV reduces with time in fatiguing muscles, the slope of the data, normalised to the initial value of the treatment, was selected as the index of fatigue [20]. CV was measured using a disposable 16-array surface electrode at a 5 mm electrode pitch (Model ELSCH016 electrode, OT Bioelettronica). Signals were sampled by a multichannel EMG amplifier (OT Bioelettronica, Torino, Italy). The EMG signals were amplified, band pass filtered (3 dB bandwidth, 10–500 Hz, roll-off of 40 dB/decade), sampled at 2048 Hz, and stored on a PC (12 bit A/D converter). VO_2_ MAX was measured using a portable O2 analyser (Model Cosmed k4B^2^) and a cycle ergometer to perform the aerobic activity.

Shoulder abduction exertion was measured using a commercial force meter (Mecmesin Advanced Force Gauge AFG-500 N) modified with an adjustable level handle attached to a platform.

### Procedure

#### Part 1 shoulder abduction MVC

The testing posture involved the participant seated, with feet firmly on the ground and their back in an upright posture, the dominant shoulder abducted to 90^0^, the elbow fully extended, and the forearm fully pronated. The handle of the force meter was adjusted to the required testing posture. Participants performed an initial warm-up which consisted of a number of repetitive shoulder movements from 0^0^ to 90^0^ abduction. Instructions to the participants informed them to abduct their pronated arm while grasping the handle of the force meter, to generate their maximum lifting effort and sustaining it for 3 sec, in line with the Caldwell regime [22]. Three trials were conducted with 5 min of rest between each treatment. The maximum result was determined as the participant’s MVC.

#### Part 2 upper trapezius CV and shoulder abduction endurance

The participants’ skin was shaven, if required, and prepared with abrasive paste and alcohol wipes to reduce skin impedance [23]. Conductive gel was injected into each space of the array electrode and secured to the skin with tape. The electrode was positioned over the upper trapezius lateral to the innervation zone, between the seventh cervical vertebra and the posterior tip of the acromion. The Innervation Zone (IZ) of the muscle was detected using a bar electrode and the position marked on the skin to position the array.

Each participant completed three static shoulder abduction endurance exertions at 0, 10, and 20% MVC. Contractions were maintained until the participant could no longer sustain the exertion. The target percentage MVC levels were calculated by the experimenter and the participants were instructed to exert the force levels visually via the real-time values on the force meter interface. The 0% MVC exertion involved holding the arm outright without exerting a lifting force on the force meter.

The treatments were randomised for each participant using Latin Square orders, which are unique orders of treatments for each participant. A 10-min resting period followed each endurance test with additional rest given if residual fatigue or discomfort was reported, as per Yassierli and Nussbaum [24].

#### Part 3 post-test measurements of the covariate VO_2_ MAX

The starting workload was estimated based on age and level of activity. The cadence was adjusted to achieve a heart rate of 60–85% of maximal capacity (i.e. 220-age) and VO_2_ MAX was measured in real time using the Cosmed software system.

### Statistical analysis

Data were presented as means and Standard Deviation (SD) of the mean. Statistical significance was set at *p* < 0.05. Statistical Analysis was performed using SPSS V 22. The Kolmogorov-Smirnov test applied to the data indicated the shoulder abduction MVC values were normally distributed, but the CV and ET data were not. The log transformation was successful in normalising these data. Independent samples t-tests were used to compare the MVC data between the young versus old age groups, and separately for males versus females. ANOVA was used to test the main and interaction effects on both CV and ET, and repeated with ANCOVA to include VO_2_ max as the covariate.

## Results

Mean shoulder abduction MVC of the young group was 73.1 N ± (27.30 SD) and mean MVC of the older individuals was 64.01 N ± (24.15 SD). This difference was not statistically significant. Mean shoulder abduction MVC for males was 87.8 N (± 21 SD) and for females 49.3 N (± 12.3 SD), which were significantly different (t-test *p* = 0.0001).

**Fig. 1:**
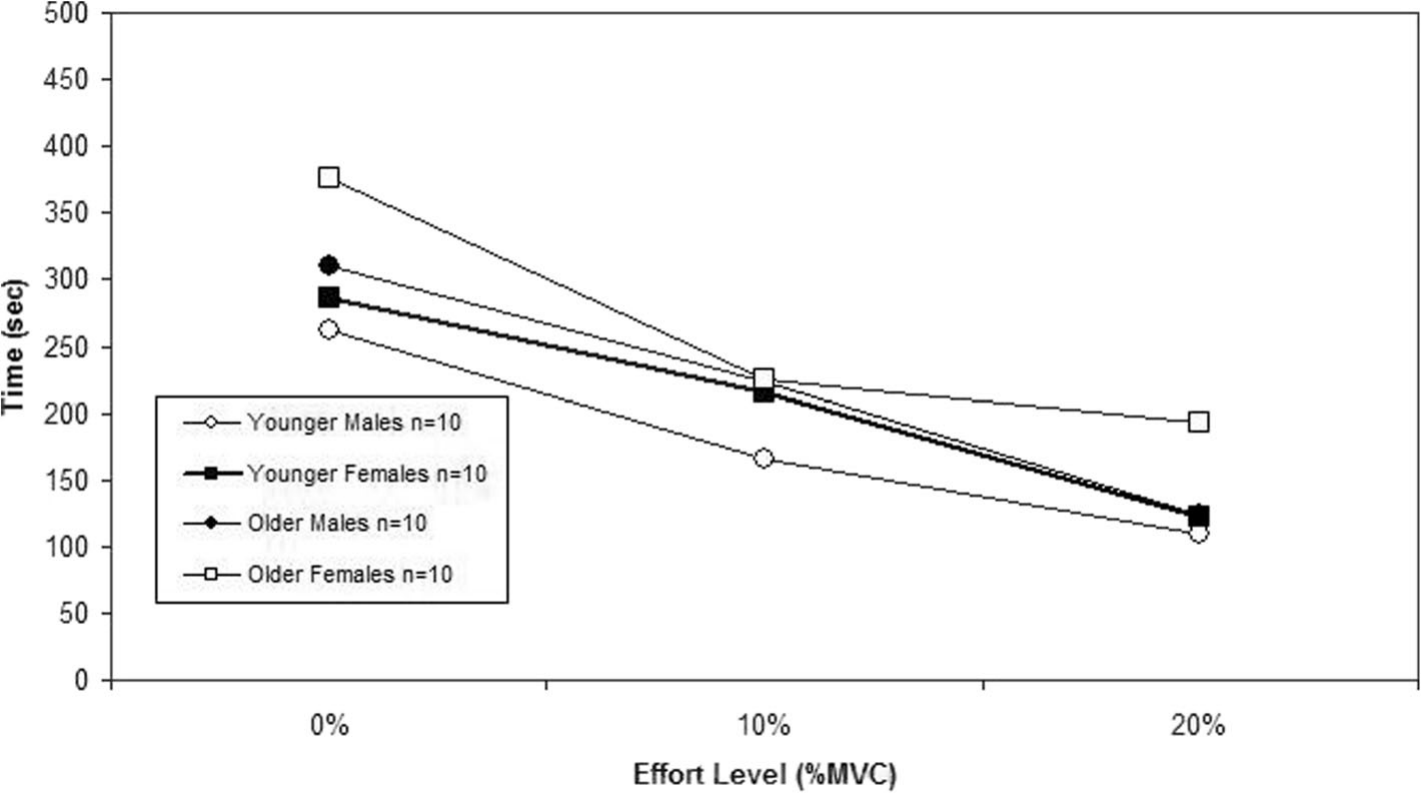
Mean endurance times illustrating the statistically significant effects for Load, Age, and the Age x Sex x Load interaction (*p* < 0.05)

Regarding endurance times, Table 1 details the results of the statistical analysis while Fig. 1 depicts the plots of the mean values by age, sex and load. Age had a highly significant effect on Endurance Time (*p* = 0.02), with higher times for the older age group. T there was no significant effect (*p* = 0.1) for sex. The three-way interaction was also significant (*p* = 0.01).

**Table 1:** Summary of ANOVA/ANCOVA main and interaction effects on endurance time

Dependent variable	Main Effects			Interaction			
	L	S	A	AxS	AxL	SxL	AxSxL
Endurance Time	0.0005**	0.1	0.021*	0.491	0.521	0.15	0.012*
Endurance Time (V0_2_ MAX as covariate)	0.0001**	0.175	0.024*	0.308	0.548	0.242	0.009**

L = Load (exertion level) S=Sex A = Age

*p < 0.05 **p < 0.01

**Fig. 2:**
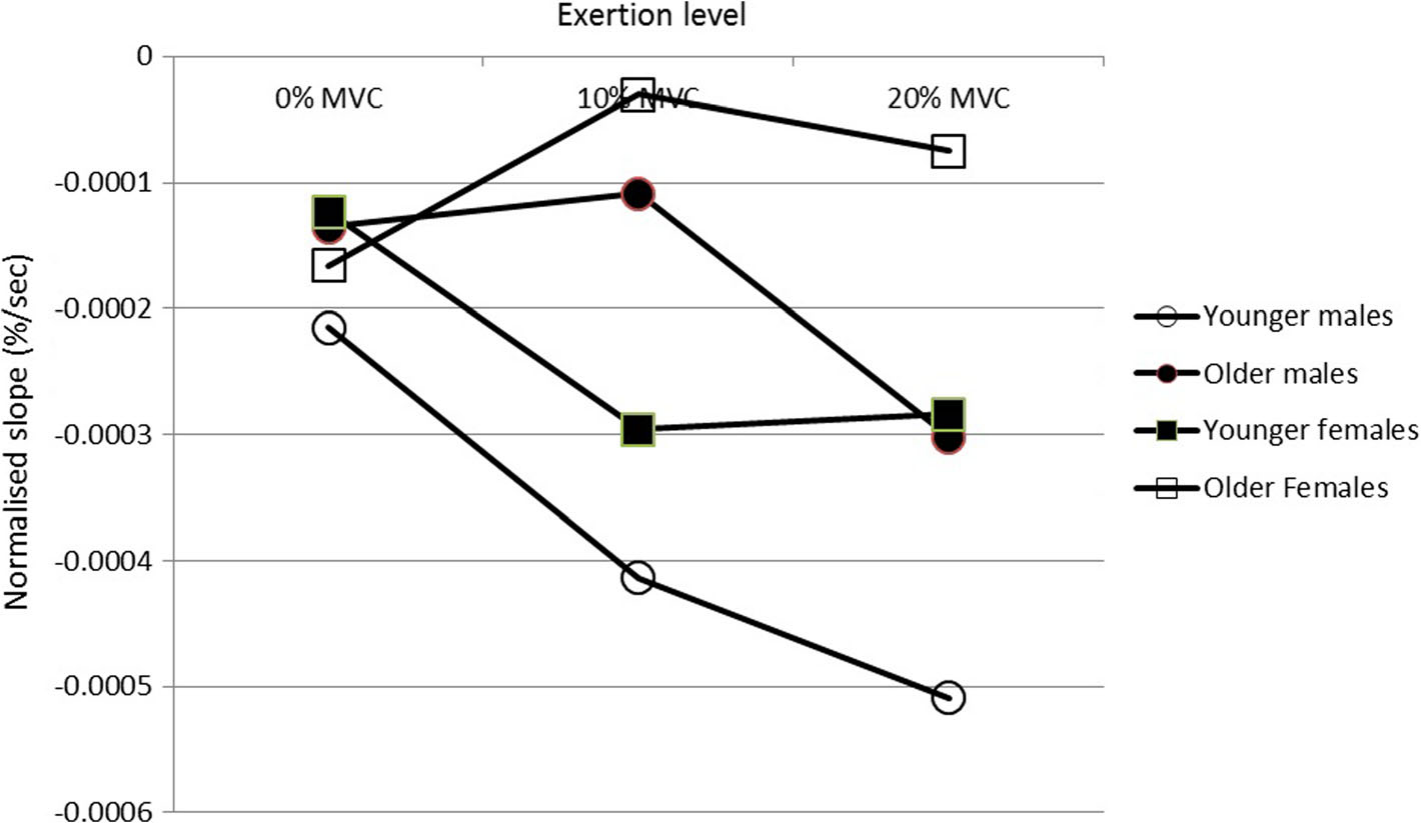
Mean normalised muscle fibre conduction velocities for the upper trapezius, by Load, Age and Sex

Regarding the upper trapezius conduction velocity data, Table 2 details the results of the statistical analysis while Table 3 and Fig. 2 detail the mean and standard deviation of the data for the experimental conditions. Age had a significant effect (*p* = 0.002) with greater slopes (higher fatigue) for the younger groups. Sex also had a significant effect (*p* = 0.016) with males showing greater slopes (higher fatigue). There was a significant Age x Load interaction (*p* = 0.035).

**Table 2:** Summary of ANOVA/ANCOVA main and interaction effects on normalised rate of change of CV

Dependent variable	Main Effects			Interaction			
	L	S	A	AxS	AxL	SxL	AxSxL
Conduction Velocity	0.049*	0.035*	0.003*	0.621	0.044*	0.187	0.845
Conduction Velocity (V0_2_ MAX as a covariate)	0.13	0.016*	0.002**	0.292	0.035*	0.154	0.827

L = Load S=Sex A = Age

*p < 0.05 **p < 0.01

**Table 3:** Mean endurance times and SD by load age and sex

Load (Exertion level)	Group	Male		Female	
		Mean	SD	Mean	SD
0% MVC	Young	262	99	285	145
	Older	310	149	377	168
10% MVC	Young	166	66	215	109
	Older	224	80	225	34
20% MVC	Young	109	31	122	48
	Older	124	39	193	25

## Discussion

The principal findings of this study are an apparent age-related improvement in muscle endurance and increased fatigue resistance for females over males. These trends have been previously observed in individual studies of age or sex for muscles groups. There are two key strengths to this study over previous fatigue studies of this nature. Firstly, it is a single experimental study of the same fatigue conditions involving both males and females of both age groups. This enables a direct comparison of the fatigue differences between these groups, which, to our knowledge, is not present in the literature for working age adults. The second key strength of this study is the measurement of muscle fatigue via muscle fibre conduction velocity for the experimental fatigue conditions tested, and for a shoulder muscle commonly associated with MSDs.

The age-related improvement in fatigue identified in this study may be due to differences in muscle fibre type compositions [25]. Aged muscles have been characterised as muscles with a type I fibre dominance [26] resulting in increased fatigue resistance [27]. Type I fibre fatigue resistance is indicated to be due to their myosin heavy chain cross bridges [28, 29]. Merletti et al. [30] detail that reduction in the motor unit firing rate also plays a role in improvement of age-related muscle fatigue resistance. Muscle fibre type differences also most likely explain the increased fatigue resistance for females. Although there is a similar distribution of fast and slow fibre types for males and females, there is, however, a significant sex-related difference in the total area occupied by type I fibres [28, 31], which increases oxidative capacity of these fibres, increasing fatigue resistance for female muscle. Fulco et al. [32] propose that female muscle has increased muscle oxidative phosphorylation, while Crowther and Gronka [33] have identified that the muscle fibres recruited first in voluntary contractions have a higher oxidative capacity than those recruited last. Sex-based differences have also been observed in muscle function [28], but explanations for these differences remain underdeveloped. In accordance with the Henneman Theory and the Cinderella Hypothesis, female muscle would contain a greater oxidative capacity prolonging fatigue. Each of these suggestions may explain the sex-related difference in the current study, however, additional tests incorporating greater loads would be advantageous in supplementing these attributes.

This study reinforces the need for clinicians and policymakers to emphasise workplace and policy-level occupational health strategies to correct the clear trends of elevated work-related injuries/symptoms for certain groups of workers. This can be achieved through group level exposure monitoring, and/or through improved workplace risk assessments/monitoring sensitivity of more vulnerable workers.

A weakness of the study is the sample size. Each group contained only ten participants, but it should be noted that this is greater than the full sample size of many other EMG-based lab studies. The sample sizes were considered adequate for statistical analysis in this experiment. However, a larger sample would clarify borderline near significant results, which may be indicative of type II errors. The testing posture, while typical of static arm positions in many construction and industrial tasks, may not represent many typical arm postures involving low levels of shoulder flexion/abduction, for example in computer work, also representing a limitation of the study. The mean age of the older participants was 59 years, which is not representative of the older general population, however it is representative of the older working age population where many people retire by the age of 65 years. Further studies should be performed with older participants than studied in this experiment to assess the wider generalisability of these findings.

## Conclusions

The overall conclusion from this research was that the older cohort exhibited decreased shoulder strength, longer endurance times, and signs of slower progression of muscular fatigue, suggesting type I fibre dominance in aged muscles. Females exhibited higher endurance times and slower progression of muscular fatigue than the males. The importance of this work is that it identified that the groups with increased fatigue resistance (older age & females) are indicated to be those typically with lower muscle strength in the working population. A key inference from the study is that when controlling for exposure, the trends in age and sex-related differences in shoulder MSD prevalence might not primarily be due to muscle fibre type related differences, but rather differences in muscle strength.
